# Late fMRI Response Components Are Altered in Autism Spectrum Disorder

**DOI:** 10.3389/fnhum.2020.00241

**Published:** 2020-06-30

**Authors:** Scott O. Murray, Tamar Kolodny, Michael-Paul Schallmo, Jennifer Gerdts, Raphael A. Bernier

**Affiliations:** ^1^Department of Psychology, University of Washington, Seattle, WA, United States; ^2^Department of Psychiatry and Behavioral Science, University of Minnesota, Minneapolis, MN, United States; ^3^Department of Psychiatry and Behavioral Sciences, University of Washington, Seattle, WA, United States

**Keywords:** excitation-inhibition balance, autism, neural excitability, functional MRI, offset response, undershoot

## Abstract

Disrupted cortical neural inhibition has been hypothesized to be a primary contributor to the pathophysiology of autism spectrum disorder (ASD). This hypothesis predicts that ASD will be associated with an increase in neural responses. We tested this prediction by comparing fMRI response magnitudes to simultaneous visual, auditory, and motor stimulation in ASD and neurotypical (NT) individuals. No increases in the initial transient response in any brain region were observed in ASD, suggesting that there is no increase in overall cortical neural excitability. Most notably, there were widespread fMRI magnitude increases in the ASD response following stimulation offset, approximately 6–8 s after the termination of sensory and motor stimulation. In some regions, the higher fMRI offset response in ASD could be attributed to a lack of an “undershoot”—an often observed feature of fMRI responses believed to reflect inhibitory processing. Offset response magnitude was associated with reaction times (RT) in the NT group and may explain an overall reduced RT in the ASD group. Overall, our results suggest that increases in neural responsiveness are present in ASD but are confined to specific components of the neural response, are particularly strong following stimulation offset, and are linked to differences in RT.

## Introduction

Autism spectrum disorder (ASD) is a behaviorally-defined neurodevelopmental disorder that includes difficulties in social communication and interaction, restricted interests and repetitive behaviors, and altered sensory responses (American Psychiatric Association, 2013; Lord and Bishop, 2015). The underlying neurophysiological basis of ASD is unknown. However, a frequently hypothesized contributor to the etiology of ASD is an increase in the ratio of synaptic excitation to inhibition (E/I) resulting from disrupted GABA-mediated inhibition (Fatemi et al., 2009; Coghlan et al., 2012; Ford and Crewther, 2016; Robertson et al., 2016). The disrupted inhibition is thought to result in neuronal hyper-excitability, an increase in noise in cortical circuits, and an overall increase in neuronal spiking (Rubenstein and Merzenich, 2003; Nelson and Valakh, 2015). One straightforward prediction of overall reduced inhibition is higher neural spike rates and thus larger amplitude neural population responses indexed with fMRI and/or ERP.

The examination of responses in the sensory cortex may be a particularly powerful approach in identifying altered neural responses in autism (Heeger et al., 2017; Robertson and Baron-Cohen, 2017). Sensory symptoms, now included in the DSM-5 diagnostic criteria, are common in ASD (Brown and Dunn, 2002; Rogers and Ozonoff, 2005; Leekam et al., 2007; Ben-Sasson et al., 2009); they persist across age (Leekam et al., 2007), are present across individuals with a range of cognitive abilities (Leekam et al., 2007), and have unique features when compared to other neurodevelopmental disorders (Rogers et al., 2003). Previous findings have shown increased fMRI (Green et al., 2013) and altered ERP (Brandwein et al., 2015; Takarae et al., 2016) responses in individuals with ASD compared to controls, that correlated with sensory over-responsiveness symptoms. Also, a relative lack of sensory-response neural habituation has been observed in ASD (Green et al., 2015; Millin et al., 2018) along with alterations in functional connectivity between sensory and saliency-based networks (Green et al., 2016). However, overall, there have not been consistent demonstrations of increased population-based responses in individuals with ASD (Milne, 2011; Dinstein et al., 2012; Haigh et al., 2015; Butler et al., 2016).

A complicating factor in characterizing response amplitude is that stimulus-evoked responses are not unitary and are composed of multiple components (Connors and Gutnick, 1990), each affected by different underlying neural circuit properties (Liu and Wang, 2001; Benda and Herz, 2003). For example, visual and auditory stimuli elicit an initial, high-amplitude transient onset response that can be observed in both spike rate (Connors and Gutnick, 1990) and fMRI (Fox et al., 2005; Uludağ, 2008) measures. The transient response is followed by a lower-amplitude sustained response that occurs for the duration of a stimulus. The magnitude of the sustained response is not only determined by stimulus properties but can be affected by mechanisms such as habituation and adaptation (Priebe and Lisberger, 2002; Priebe et al., 2002) which we have shown are disrupted in ASD (Millin et al., 2018).

In addition to transient and sustained increased neural responses that occur while a stimulus is present, there are also well-defined stimulus-offset responses that occur upon removal of sensory input. The offset response can take different forms; it can manifest as an increase above the sustained neural response (Harms and Melcher, 2003; Qin et al., 2007; Kopp-Scheinpflug et al., 2018), or as suppression of neural responses below spontaneous baseline levels (Shmuel et al., 2006). In the fMRI response, this latter suppression appears as an “undershoot” in the return to baseline after a stimulus has been removed and is believed to reflect neural inhibition. Simultaneously measured EEG-offset response amplitudes that are tied to inhibition are correlated with fMRI undershoot amplitudes (Mullinger et al., 2013, 2017). Also, simultaneous spike-rate and fMRI measurements in monkeys have shown that stimulus-offset spike-rate suppression is related to the fMRI undershoot (Shmuel et al., 2006), strengthening the link between the fMRI undershoot and inhibitory processes. This relationship between the fMRI undershoot and neural inhibition is of particular interest in ASD given the possible role of disrupted inhibition (Coghlan et al., 2012) and its potential impact on increasing neural noise (Leventhal et al., 2003). Indeed, previous fMRI findings have demonstrated a disrupted post-stimulus undershoot in individuals with schizophrenia (Hanlon et al., 2016), a disorder that also has been associated with altered inhibitory processing (Lewis et al., 2005; Hasan et al., 2012; Stan and Lewis, 2012; Hoftman et al., 2015; Foss-Feig et al., 2017). We know of no such investigation in ASD testing whether the undershoot may be disrupted or absent.

To evaluate changes in stimulus offset responses in ASD and to compare them to more typically measured transient responses, we used an experimental procedure that elicited neural responses simultaneously in multiple sensory areas and the motor system in young adults with ASD compared to neurotypical (NT) controls. This dataset was used previously to specifically characterize sustained responses and adaptation in ASD, primarily in the visual and auditory cortex (Millin et al., 2018). In our previous publication, while we noted the presence of a difference in the fMRI undershoot between our ASD and control groups, this component was not further characterized. Here, we expand our investigation to brain-wide analyses with particular emphasis on the fMRI offset-response and report five novel findings. First, we show that fMRI offset response is a unique and separable component—its magnitude is independent of the magnitude of transient response components. Second, individual differences in fMRI offset response are strongly correlated between brain regions, suggesting that such offset response differences are pervasive and brain-wide. Third, we show that in addition to offset-response decreases in the fMRI response (i.e., signal reduction below baseline, also known as “undershoot”) there are also regions that display transient, offset-response increases, the magnitude of which differs between ASD and controls. Fourth, we demonstrate a relationship between offset-response magnitude and reaction time (RT) that may explain RT decreases in ASD. Fifth and finally, we provide a descriptive model that allows us to estimate what underlying differences in neural inhibition might look like that may be driving group differences in offset-response magnitude. Overall, we demonstrate a lack of an fMRI undershoot in individuals with ASD which may be attributable to disrupted neural inhibition.

## Materials and Methods

### Participants

Participants included 21 right-handed individuals with (ASD; six females) and 33 right-handed neurotypical (NT) subjects (15 females; see Supplementary Figure S3 for an analysis that equates males and females in the ASD and NT groups). Three ASD subjects (one female) and one NT (female) were removed from analyses due to excessive head motion and/or poor behavioral performance (criteria detailed below); *thus, the final number included 18* *ASD and 3*2 *NT participants*. All subjects had normal IQ (WASI-II Full-Scale IQ of at least 80), and normal or corrected-to-normal vision. Groups were of equal IQ and ages (mean IQ of subjects with autism: 112; NT subjects: 114; *t*_(48)_ = 0.33, *p* = 0.74; mean age of subjects with autism: 23 years; NT subjects: 24 years; *t*_(48)_ = 0.333, *p* = 0.74). All subjects provided written informed consent to participate. The Institutional Review Board of the University of Washington (UW) approved the research protocol. Subjects with ASD met diagnostic criteria for ASD on the Autism Diagnostic Interview-Revised (ADI-R; Rutter et al., 2003), the Autism Diagnostic Observation Schedule—2nd Edition (ADOS-2; Lord et al., 2012) and according to expert clinical judgment using DSM-5 (American Psychiatric Association, 2013) criteria.

**Figure 1:**
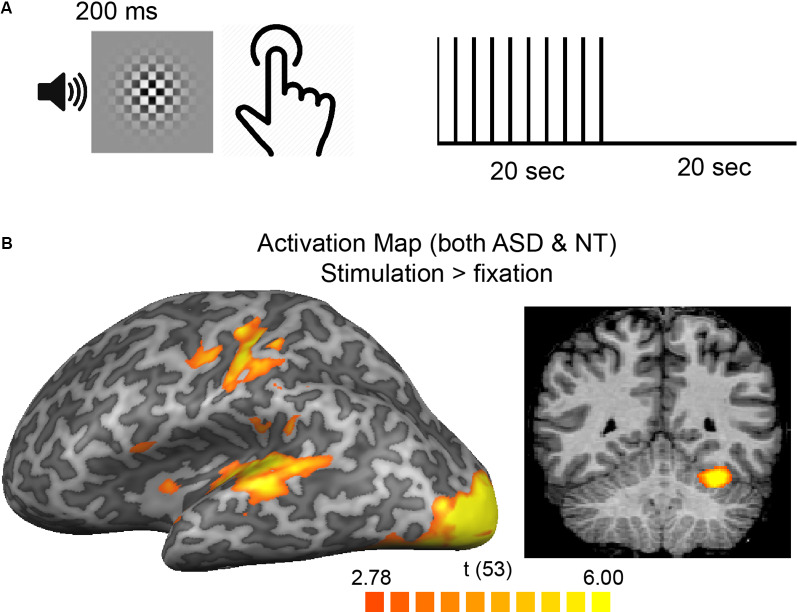
**(A)** Participants pressed a button in response to a brief, simultaneous presentation of an auditory-visual stimulus. Stimuli were presented, on average, every 2-s in 20-s blocks separated by 20 s of rest. **(B)** A group average [both autism spectrum disorder (ASD) and neurotypical (NT)] activation map overlaid on the cortical surface of the left hemisphere (left) and a single coronal slice showing cortical and cerebellar regions with increased responses to a stimulus vs. rest blocks.

**Figure 2:**
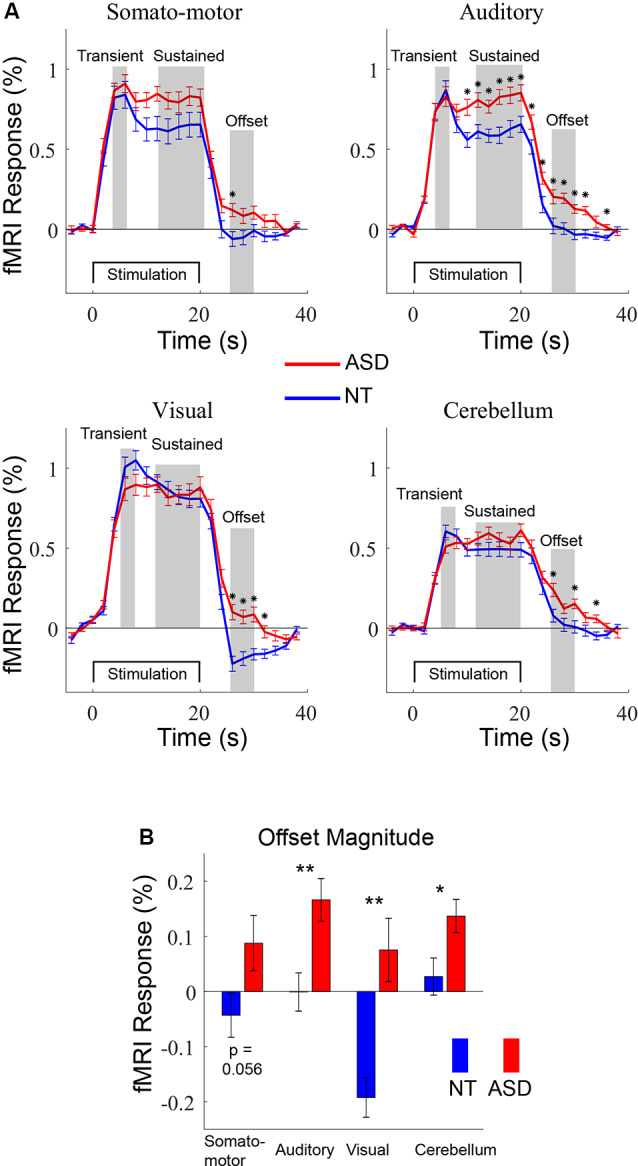
**(A)** Averaged fMRI timecourses in four regions-of-interest (ROIs) for ASD (red) and NT (blue) participants. The time courses were used to guide the definition of three response windows: transient, sustained, and offset. **(B)** Offset magnitudes for NT and ASD participants in the four ROIs. *p < 0.05. **p < 0.01; error bars = SEM.

**Figure 3:**
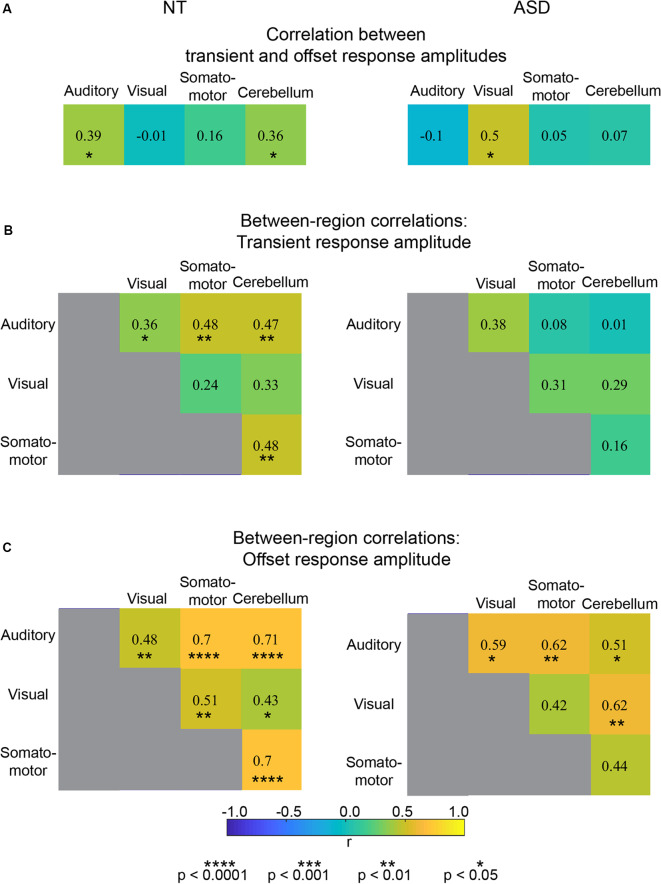
**(A)** Correlation values for individual differences in transient and offset amplitude within four ROIs. **(B)** Correlation values for between-region individual differences in transient response amplitude. **(C)** Correlation values for between-region individual differences in offset response amplitude.

**Figure 4:**
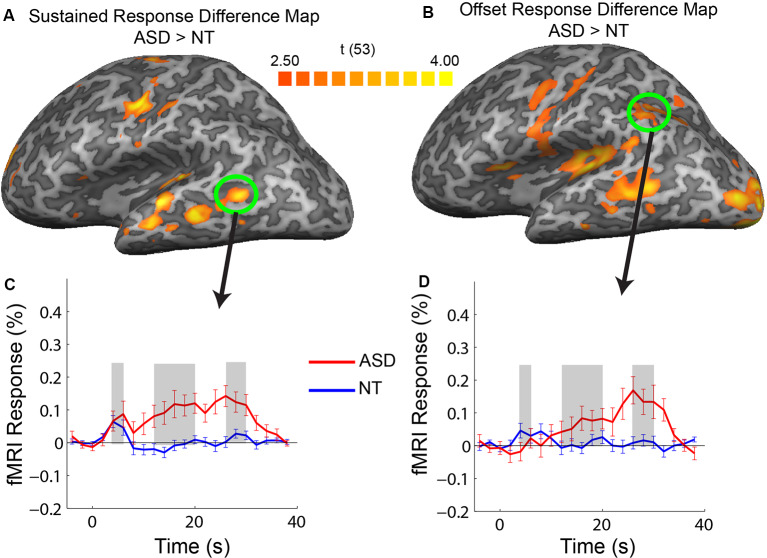
Difference maps showing regions of increased response in ASD vs. NT in sustained **(A)** and offset **(B)** response amplitude. Averaged timecourses from example ROIs that did not correspond to regions strongly activated by the stimulus, in the posterior, middle temporal region **(C)** and posterior parietal **(D)** regions.

**Figure 5:**
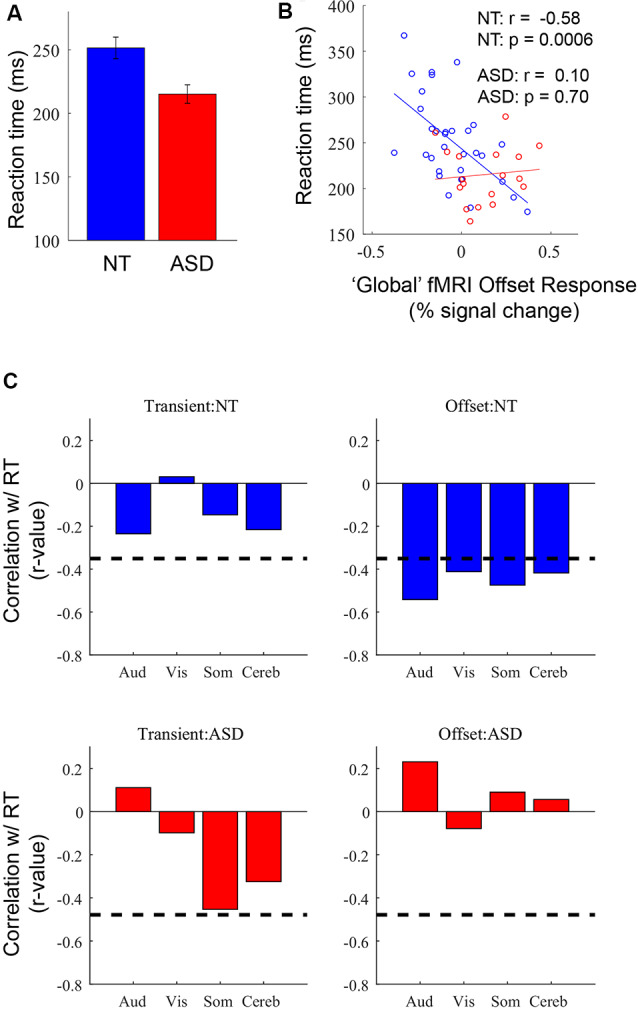
**(A)** Average reaction time (RT) in NT and ASD participants. **(B)** Correlation between RT and offset response amplitude averaged across the four ROIs in NT (*n* = 32; blue) and ASD (*n* = 18; red) participants. **(C)** Correlations between transient and offset response magnitudes with RT in each of the four ROIs. Dashed line represents *p* = 0.05.

**Figure 6:**
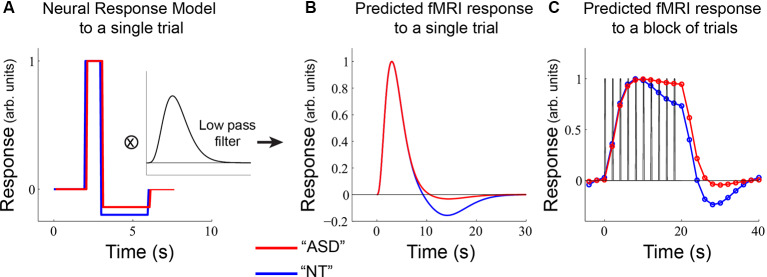
A model of potential underlying differences in neural response in sensory-motor regions. **(A)** ASD and NT participants were modeled as having the same increase in neural response but differences in neural offset responses. This neural response was passed through a low-pass filter to obtain the predicted fMRI response to a single stimulus event **(B)**. **(C)** A predicted fMRI response was obtained by convolving the function in **(B)** with a block of 10 trials (as used in the main experiment).

**Figure 7:**
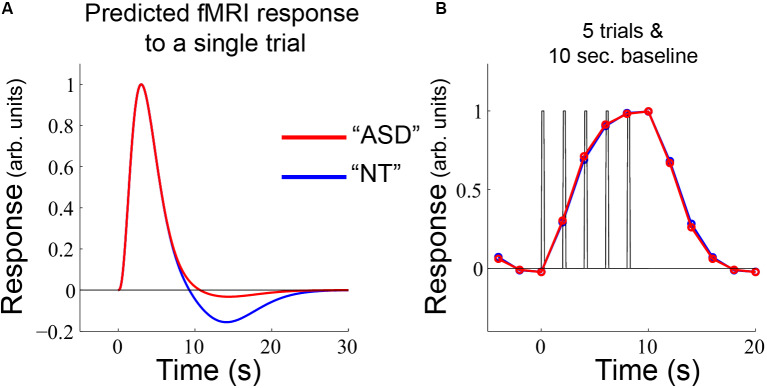
**(A)** The same single-trial model as used in Figure 6 was applied to **(B)** an alternative experimental design, which yields nearly identical predicted fMRI timecourses for NT and ASD.

Other data from these same (or subsets of these) subjects have been described elsewhere (Millin et al., 2018; Murray et al., 2018; Schallmo et al., 2018, 2019; Kolodny et al., 2020a,b). Most relevant is the Millin et al.’s (2018) study. The current manuscript includes the same fMRI data as Millin et al. (2018), plus data from four additional NT subjects that were collected and analyzed after publishing (Millin et al., 2018). We used the same data exclusionary criteria as Millin et al., 2018 (explained below) except that we did not include a time-series noise analysis that we deemed less important for characterizing offset-response magnitude. As described in the introduction—while the underlying dataset itself overlaps with Millin et al. (2018)—the analyses presented here are novel and distinct and focus on separate components of the fMRI response. Specifically, all whole-brain analyses are unique to the current article (Figures 1, 4), as are the analyses of the somatomotor and the cerebellum regions-of-interest (ROIs; Figure 2A, upper-left, and lower-right panels). The definition of a post-stimulus time window and quantification of neural responses in that window are also presented here for the first time (Figure 2B). Correlations within and between ROIs and time-windows (Figure 3) are also new. The analysis that is most closely related to those presented in Millin et al. (2018) yet still distinct, is the analysis of the timecourses in the visual and auditory ROIs presented in Figure 2A (upper-right and lower-left panels). While related, the analysis in the current article has been done point-by-point to examine differences throughout the whole time course in greater detail, whereas in Millin et al. (2018) group comparisons were conducted on averaged responses in the transient- and sustained-responses time windows. Also, Figure 5A graphically presents a difference in RT that was reported in Millin et al. (2018); all follow-up analyses on RTs correlations (Figures 5B,C) are new. The modeling section (Figures 6, 7) is also unique to the current article and describes a new model, that is different than the one reported in Millin et al. (2018) featuring group differences in the undershoot magnitude, as opposed to group differences in the degrees of neural adaptation.

### MRI Acquisition

Scans were acquired with a Philips Achieva 3T MRI system with a 32-channel high-resolution head coil. A T1-weighted MPRAGE structural scan was acquired at the beginning of the scan session, followed by three functional gradient-echo EPI scans with axial orientation (30 slices with 3 mm in-plane resolution and 0.5 mm gap, 2 s TR, 25 ms TE, 79° flip angle, A-P phase-encode direction). A single TR EPI scan with opposite phase-encoding direction (P-A), but otherwise identical to those above, was acquired for use in correcting geometric distortions. Each subject underwent a single scanning session, lasting approximately 1 h (the scan session also included the acquisition of spectroscopy data for a separate experiment).

### Stimuli

Stimuli were presented using Presentation 14.9 software running on a Windows XP computer. Images were projected onto a screen behind the subject’s head *via* either an Epson Powerlite 7250 or an Eiki LCXL100A projector (following a hardware failure), both operating at 60 Hz and with linearized luminance profiles. Subjects viewed the projected images using a mirror positioned above their eyes, for an effective viewing distance of 66 cm. The sound was delivered at 44.1 kHz using MRI compatible earbuds (S14, Sensimetrics). Subjects wore protective ear muffs over the earbuds to attenuate acoustic noise from the scanner. Before scanning, subjects verified that the auditory stimulus was presented at an audible and comfortable volume.

The visual stimulus consisted of a Gaussian-windowed (with FWHM of 2.75° visual angle; the approximate visible size of 5.2°), full-contrast checkerboard (check size of 0.4°) image presented on a uniform background accompanied by audio white noise. Visual and auditory stimuli were presented simultaneously in individual 200 ms (12 video frames) trials in a blocked design, with the stimulus presented 10 times within each stimulus block. For ease of description, each stimulus event is referred to as a “trial.” Every 20 s of stimulus presentation is referred to as a “stimulus block.” Stimulus blocks were of two types: fixed-interval and randomized-interval. In fixed-interval blocks, the stimulus trials were separated by 1,800 ms, resulting in a stimulus presentation every 2 s. In randomized-interval blocks, the inter-stimulus interval was a random value drawn from a uniform distribution bounded by 800 ms and 2,800 ms. Stimulus blocks alternated with 20-s long passive fixation (rest) blocks. Subjects were asked to press a button with their index finger on their dominant hand as quickly as possible in response to each stimulus presentation. For the current experiment that is focused on offset response magnitudes, the timing conditions of the stimuli are less relevant. Further, we did not observe block-type effects or group interactions, as described in a supplemental analysis in the initial part of the “Results” section. Considering both of these factors, the fixed-interval and randomized-interval blocks were analyzed together for all analyses. We note that separate analyses of these conditions with a subset of the current subjects were presented elsewhere (Millin et al., 2018). A fixation cross appeared at the center of the display whenever the stimulus was off. Each subject completed three runs comprised of eight stimulus blocks and nine rest blocks each. Subjects were instructed to use the index finger of their right hand to press a button as quickly as possible following the appearance of each stimulus. Subjects practiced the task during a mock scan session before scanning.

### MRI Data Analysis

Data were preprocessed using BrainVoyager QX version 2.8 (Brain Innovation, Maastricht, The Netherlands) software. EPI data were motion-corrected, corrected for distortion due to magnetic field inhomogeneities, high-pass filtered (cutoff = 2 cycles/scan), coregistered to the AC-PC-aligned T1 structural scan, and transformed to Talairach space (Talairach and Tournoux, 1988). Multiple analysis strategies were employed including ROI-based and whole-brain approaches. First, to identify regions that were active in response to the combined finger-press/visual/audio stimulus presentation (“stimulus blocks”), a standard general linear model was performed across all participants (ASD and NT) identifying voxels that had increased activity to the stimulation blocks relative to the passive fixation blocks (results shown in Figure 1B). Specifically, a boxcar predictor for the entire stimulus block was convolved with a double gamma hemodynamic response function (HRF; parameters: time-to-response peak = 5 s; time-to-undershoot peak = 15 s; response-undershoot-ratio = 6) and used as the predictor in a general linear model that was fit to the timecourse of each voxel. The activation map identified four primary ROIs: somatomotor (along the central sulcus), auditory (superior temporal region), visual (near the occipital pole), and cerebellum (right dorsal region). Subsequently, ROIs corresponding to these four areas was defined on a subject-by-subject basis to account for differences in anatomy that exist between individuals. For each subject, the same GLM approach was used. The resulting activation maps from the *t*-statistic for the model fit were initially thresholded at *p* < 0.05 (whole-brain, voxel-wise corrected for multiple comparisons). ROIs were selected manually from the most significant areas of activation near visual (left and right hemisphere), auditory (left and right hemisphere), somatomotor (left hemisphere) cortices, and right dorsal cerebellum yielding six total ROIs for each subject (left and right hemispheres were averaged yielding the four final ROIs; separate between-hemisphere analyses were initially conducted and no differences within or between groups were observed). For the early visual ROI, the cluster nearest the occipital pole, in line with the calcarine sulcus, was selected. The 20 most significantly activated voxels in the cluster defined the final ROI in each region. For some ROIs in some subjects, fewer than 20 voxels met the threshold criteria; in these cases, the threshold was relaxed until 20 voxels could be selected. If no obvious cluster of voxels was present after the threshold was lowered, the ROI was excluded. This resulted in 1 ASD subject without an identifiable auditory ROI and 1 NT and 1 ASD subject without an identifiable somatomotor ROI.

For ROI-based analyses (Figure 2), average timecourses across the 20 voxels in each ROI were determined for each run. Percent-transformed timecourses were then calculated for each block. First, for each stimulus block, we extracted 22 timepoints corresponding to −4 s before stimulus onset to 38 s after stimulus onset (TR, the sampling rate, was 2 s). Then we converted the values to percent signal change relative to the mean value of timepoints −4, −2, and 0 (reflecting the best-estimate of “baseline” before stimulus onset). Specifically, each timecourse was normalized by subtracting and dividing by the mean of the pre-stimulus TRs and multiplying by 100. The resulting block timecourses were then averaged over blocks. Blocks that did not meet the criteria detailed below for head motion and task performance were excluded before averaging. Data for a given block was excluded due to head motion if the subject’s head moved more than 0.9 mm between two successive TRs (frame-wise displacement >0.9 mm; Siegel et al., 2014) up to and including 8 TRs before or 1 TR after the stimulus block. A block was excluded based on task performance if it contained any misses (failure to press the button after a stimulus appearance) or more than one false alarm (more than one button press after the appearance of a stimulus). If more than half the blocks were excluded, all data for the subject were excluded. These behavioral and head-motion data exclusionary procedures resulted in the removal of four subjects (three ASD for head motion and one NT for behavioral performance). An analysis of head-motion was performed on included data, after the removal of blocks for excessive head motion; see Supplementary Figure S4.

Analyses were performed on separate components of the fMRI response. Components were initially identified through visual inspection of the grand-average (across both ASD and NT) timecourses (Figure 2A) and defined as “transient” (4–6 s post-stimulation onset), “sustained” (12–20 s post-stimulation onset), and “offset” (26–30 s post-stimulation onset; 6–10 s after the stimulation block ended). Subsequent whole-brain analyses were performed on Talairach normalized data. The mean value for each voxel in the above time windows was calculated. A between-groups (2-sample) *t*-test was performed for each voxel, to identify regions in which fMRI response components differed between groups. Resulting thresholded significance maps were visualized on the cortical surface.

### Modeling

Modeling the fMRI timecourse was performed using MATLAB and a combination of custom code and the “hrf.m” function in BVQXtools^1^. The goal of the modeling was to provide a qualitative description of the types of neural responses that could potentially contribute to the observed fMRI responses. Quantitative fitting between the model and the fMRI timecourse was not appropriate; the number of equivalent solutions does not have a unique set of parameter values. Instead, we chose parameter values that seemed biologically plausible and produced good qualitative approximations of the fMRI timecourses.

For Figure 6, we used a single-gamma (“Boynton”) filter with a peak time of 5 s and convolved it with a box-car estimate of the single-trial neural response (Figure 6A) with a maximum value of 1.0 for 1.0 s and a minimum value of −0.2 for the NT response and −0.14 for the ASD response that lasted 3.0 s. It should be noted that the output of this convolution (Figure 6B) could also be obtained using a typical “double-gamma” HRF function that includes both a positive and negative component. The point of Figure 6 was to show the consequences of a relatively small change in the underlying neural offset response on the fMRI response (Figure 6C).

## Results

### fMRI Analysis: Regions-of-Interest

Participants pressed a button in response to a brief, simultaneous audio-visual stimulation during stimulation blocks, which alternated with rest blocks (Figure 1A). A whole-brain GLM comparing stimulation blocks to rest blocks across all participants resulted in higher responses in expected regions (Figure 1B) including posterior occipital regions that are known to process visual information, temporal regions that process auditory information, regions near the central sulcus involved in finger movement and somatosensation, and the cerebellum. A second GLM comparing the ASD and the NT groups tested the basic prediction of the neural excitability hypothesis—that fMRI responses are higher for ASD than NT participants. No significant differences (in either a positive or negative direction) in any voxels were observed. Thus, taking into account the entire stimulation block—as typically analyzed—there were no differences in fMRI response magnitude between groups.

To compare different components of the response we defined ROIs in primary auditory, visual, motor, and cerebellar areas for each subject and then extracted averaged fMRI timecourses for the ASD and NT controls (Figure 2A). For both groups, there were distinct components of the response including an initial high amplitude transient response followed by a lower amplitude sustained response. To visualize group differences in the timecourses, *t*-tests were performed at each timepoint and labeled with “*” to denote *p* < 0.05. The transient response—which is likely the most straightforward measure of neural excitability—did not include any timepoints that were different between the ASD and NT groups in any of the four ROIs. There was a difference in multiple time points during the sustained response in the auditory ROI, as described in detail elsewhere (Millin et al., 2018). We note our relative lack of statistical power (*N* = 18 participants with ASD) likely limits the statistical significance of the trend-level difference between groups in the somatomotor ROI. The most prominent difference in the timecourses occurred after the stimulation ends, in the offset response. This is most clearly apparent in the visual ROI; in NT participants the fMRI signal goes below baseline, peaking approximately 6–8 s after the termination of the sensory-motor stimulation before returning to baseline levels. In ASD participants, this “undershoot” is almost completely absent. Note that analyses were initially performed separately on the two stimulus timing conditions (fixed- and randomized-intervals) and there were no main effects (group or timing conditions) or interactions (see Supplementary Figure S1). Thus, all subsequent analyses average across timing conditions to increase signal-to-noise, reduce multiple comparisons, and to simplify descriptions of results. To quantify the differences in offset magnitude, we averaged the fMRI signal 26–30 s after stimulus onset (6–10 s after offset; Figure 2B). Significant differences between the ASD and NT groups were observed in the auditory (*t*_(47)_ = 3.00, *p* < 0.001), visual (*t*_(48)_ = 4.18, *p* < 0.001), and cerebellum (*t*_(47)_ = 2.13, *p* < 0.05) ROIs. The somatomotor ROI was just above statistical significance levels (*t*_(46)_ = 1.96, *p* = 0.056) again possibly the result of relatively low statistical power due to modest sample size.

Using an individual differences approach, we addressed the degree of independence of the transient and offset response components. First, we assessed whether individual differences in transient response magnitude were associated with individual differences in offset response magnitude (Figure 3A). For both NT and ASD groups, correlation strengths were either non-significant (NT: Visual, *r* = −0.01, *p* = 0.94 and Somato-motor, *r* = 0.16, *p* = 0.38, ROIs; ASD: Auditory *r* = −0.10, *p* = 0.71, Somato-motor, *r* = 0.05, *p* = 0.86 and Cerebellum, *r* = 0.07, *p* = 0.78) or of modest strength (NT: Auditory, *r* = 0.39 *p* = 0.03, and Cerebellum, *r* = 0.36, *p* = 0.04; ASD: Visual, *r* = 0.50, *p* = 0.04). Thus, overall, whether a person has a relatively large or small transient response in a particular region does not consistently or strongly predict whether they have a relatively large or small offset response in that same region.

Next, we addressed whether individual differences in transient response magnitude in one region were associated with individual differences in transient response magnitude in another region (Figure 3B). Again, for both groups, the association was weak-to-modest suggesting that transient response magnitudes for a particular region are relatively independent of other brain regions; an individual could, for example, have a large transient response in the visual ROI and a small transient response in the auditory ROI. Correlation strengths ranged in the NT group from *r* = 0.24, *p* = 0.20 (between visual and somatomotor ROIs) to *r* = 0.48, *p* = 0.006 (between the somatomotor and cerebellum ROIs). In the ASD group the range was from *r* = 0.01, *p* = 0.97 (between auditory and cerebellum ROIs) to *r* = 0.31, *p* = 0.23 (between visual and somatomotor ROIs).

Finally, we examined whether individual differences in offset response magnitude in one region were associated with individual differences in offset response in another region (Figure 3C). Strikingly, for both NT and ASD groups, the correlation strengths between all ROI pairs were significant (except one in the ASD group that was trend-level) and the majority of the association strengths ranged from moderate to very strong. In the NT group the correlation strengths ranged from *r* = 0.43, *p* = 0.02 (between the visual and cerebellum ROIs) to *r* = 0.70/0.71, *p* < 0.00001 (between the auditory and somatosensory and cerebellum ROIs). In the ASD group the correlation strengths ranged from *r* = 0.42, *p* = 0.10 (between the visual and somatosensory ROI) to *r* = 0.62, *p* = 0.01 (between the auditory and somatosensory ROI). Thus, if an individual has a large (or small) offset response in one region they will likely have a large (or small) offset response in another region. Taken together, these results suggest that individual differences in offset response magnitude are relatively independent of transient response magnitude (Figure 3A) and potentially represent a brain-wide individual characteristic (Figure 3C). As our modeling will demonstrate below, the fMRI sustained response magnitude can be affected by neural transient response magnitude, offset response (undershoot) magnitude, or some combination of two. Thus, correlation values between the sustained response and offset (or transient) response are more difficult to interpret. For completeness, these correlations are reported in Supplementary Figure S2 and show, as expected, values that are of intermediate magnitude.

### fMRI Analysis: Whole-Brain Analysis

Our ROI analysis presented above was restricted to cortical regions that had increased responses across the entire stimulation period. In a subsequent exploratory analysis, we identified regions with response magnitude differences between groups confined to the transient, sustained, and offset temporal windows. We extracted the timecourses for each voxel in the brain and created group-difference maps for each of the three temporal response windows. There were no voxels with a significant increase in the transient response window for the ASD vs. NT subjects. There were, however, multiple cortical areas in the ASD participants that had increases in response magnitude in the sustained and offset response windows relative to the NT participants. Specifically, increases in the sustained temporal window in ASD were primarily confined to the motor and auditory regions that were activated by the stimulus, consistent with what was observed in the ROI analysis. Also, there were regions in the middle-temporal sulcus with an increased sustained response in ASD that did not correspond to regions strongly activated by the stimulus (Figure 4A, green circle).

The most widespread increase in response in ASD relative to NT occurred after termination of the stimulation block during the offset response window. Increases in the offset window were observed in all regions activated by the stimulus (motor, auditory, and visual) along with regions in the posterior parietal cortex and the middle temporal sulcus. As previously mentioned, the ASD increase in offset response in sensory regions appears to be associated with a lack of an fMRI undershoot (e.g., see Figure 2A, visual). However, an inspection of offset differences in regions outside of primary sensory areas (e.g., in the middle temporal sulcus and posterior parietal cortex) reveals a different pattern to the timecourse. Specifically, there is little or no response above baseline in these regions in the NT participants; however, in ASD participants, there is a progressive increase in response followed by a positive-going response during the sensory offset time window. Thus, the increase in offset response in ASD in these regions appears to have a different neural origin, unrelated to a difference in the more negative-going undershoot.

### Reaction Time

In our previous analyses of these data (Millin et al., 2018) we reported a group difference in RT (RTs) for the button press—RTs were shorter for the ASD than NT group (Figure 5A)—but we could not provide a potential mechanistic explanation. Here, we speculated that since the fMRI offset response may be partially driven by inhibitory processing there might be a relationship between individual differences in offset response magnitude and RTs; specifically, if a stronger negative offset reflects greater inhibition, this may be associated with longer RTs. First, to simplify the analysis, we calculated a single, “global” measure of offset response magnitude for each subject by averaging the offset response in the four ROIs identified in Figure 2. Next, we correlated individual differences in global offset response magnitude with individual differences in RT. In the NT group, more negative offset responses were associated with longer RTs (Figure 5B; *r* = −0.58, *p* = 0.0006). In other words, individuals with a stronger undershoot—and, presumably, stronger brain-wide inhibition—have longer RTs. In the ASD group, there was no relationship between individual differences in offset response magnitude and RTs (*r* = 0.10, *p* = 0.70). This initial observation raises several questions. First, we assessed whether the neural processing associated with the undershoot builds up across trials such that RT is slowed by an accumulation of inhibition over a stimulus block. This was assessed by examining the correlation when only using the RT of the first trials in a block. These trials occur after a 20 s rest period and thus are unlikely to have any previous-trial effects. We found that the pattern of correlations is the same when only using RT to the first trial: a strong correlation in the NT group (*r* = −0.62, *p* < 0.0001) and no significant correlation in the ASD group (*r* = −0.17, *p* = 0.51). Thus, it is unlikely that RT is influenced by previous-trial effects. Second, it might seem plausible that motor-related ROIs might have a stronger relationship with RT. Also, it might be possible that any metric of neural response magnitude may be associated with RT, not just the offset-response. To assess both the ROI-specificity and the fMRI-response specificity, we examined correlations between each of the four ROIs separately for the transient and offset responses (Figure 5C). The offset-response, but not the transient response, in the NT group for all four ROIs were significantly correlated with RT. Thus, the relationship to RT appears to be independent of ROI but specific to the offset response. Based on the above pattern of results, we suggest that the longer RTs in NT individuals are linked to stronger stimulus offset responses, a relationship that may reflect stronger inhibition across individuals. However, this relationship is not observed in participants with ASD, consistent with disrupted inhibition. This speculation is described in more detail in the “Discussion” section.

### Modeling

Finally, we modeled plausible underlying neural responses that could give rise to our observed differences in NT and ASD fMRI responses in the ROI analyses (Figure 2). Broadly, this pattern consisted of an equivalent transient response, an emerging difference in the sustained response, and a prominent difference in the offset response. We considered a simple neural model for each brief trial (Figure 6A) that assigned equal neural response amplitudes to the ASD and NT groups. Following the increase in neural response, we modeled a stimulus-offset undershoot response (a neural response that goes below baseline after stimulation ends) that was slightly larger in the NT participants. We then convolved this hypothetical trial-based neural response with a low pass filter that approximates the hemodynamic lag in the fMRI response. This yielded a predicted trial-based fMRI response that closely resembles the well-known two-component hemodynamic response function (HRF; Figure 6B). We then convolved the single-trial response with our experimental design: 10 trials (2 s each) and a 20 s baseline period (Figure 6C). This yielded a predicted fMRI response that was similar to what was observed in primary sensory areas (e.g., compare Figure 6C to Figure 2A). Importantly, the model demonstrates that differences in sustained response can emerge purely from differences in the undershoot magnitude, due to an accumulation of negative responses over time. We note, however, that there are likely multiple other factors contributing to sustained response magnitude such as differences in adaptation, as we have previously shown (Millin et al., 2018).

## Discussion

Disrupted neural inhibition, which is thought to lead to an increase in neuroexcitability and an increase in neural noise (Rubenstein and Merzenich, 2003; Nelson and Valakh, 2015), is an often-cited potential neurophysiological mechanism for ASD (Fatemi et al., 2009; Coghlan et al., 2012; Ford and Crewther, 2016; Robertson et al., 2016). We examined an fMRI component that is associated with stimulus offsets—that manifests as an undershoot in the return of the fMRI response to baseline—and may rely on a specific inhibitory neural process. We observed widespread changes in stimulus offset responses in ASD, some of which could clearly be attributed to a lack of an undershoot and thus are consistent with disrupted inhibition. The potential disruption of inhibition does not appear to be ubiquitous, as other response components are not affected. For example, the transient response—the initial response to the onset of a stimulus block which presumably is most sensitive to intrinsic differences in neural excitability—was equivalent between groups in all cortical regions. Only in later response components and, in particular, after the stimulation block was over, did we observe larger amplitude fMRI responses in ASD.

Recent findings have suggested the fMRI undershoot reflects inhibitory neural processes that occur after the removal of the excitatory drive (Mullinger et al., 2013, 2017). There are, however, differences in the stimulus paradigm used in the current experiment compared with previous studies (e.g., Mullinger et al., 2017). For example, our “stimulation period” involved relatively brief, transient stimulus presentations over 20 s. Previous experiments examining hemodynamic properties such as the undershoot have used continuous stimulation such as flickering checkerboards. Since our stimulation paradigm may induce different neural response patterns than the more continuous stimulation protocols used in previous experiments, we are not able to definitively conclude the inhibitory mechanisms underlying the lack of undershoot in our ASD participants. Future research that uses stimuli matched to these earlier studies and/or also measures associated EEG responses will be required.

Absent these additional experiments, one strong suggestion that the undershoot we observed in our experimental paradigm has a neuronal origin is the strong relationship between undershoot magnitude and finger-press RT in the neurotypical control subjects. This observation is consistent with previous observations of a relationship between negative-BOLD magnitude and sensory detections thresholds (Kastrup et al., 2008). The directionality of the relationship between RT and undershoot size may, initially, seem surprising. However, our interpretation is that undershoot magnitude is related to the strength of overall cortical inhibition; thus, stronger undershoots imply more cortical inhibition and a slowing of the initiation of a motor response. We speculate that in the ASD participants, the inhibitory process related to the fMRI undershoot is disrupted (or does not exist). A difference in inhibitory functioning, resulting in small undershoot magnitude, may serve as a possible basis for the overall reduction in RT for the ASD participants. Thus, at least within the narrow confines of this experimental paradigm, the lack of an undershoot (and its implied lack of inhibition) results in a behavioral advantage.

The possibility that there could be a neural basis for the lack of an undershoot in ASD may have significant implications for understanding the neural circuits that are disrupted and may provide specificity for long-standing speculation that disrupted inhibition contributes to ASD. Despite the evidence for disrupted inhibition, we note that this same cohort of ASD and NT subjects did not have any differences in GABA concentration in some of the same brain regions that exhibited differences in undershoot magnitude (e.g., visual, auditory, and somatomotor ROIs) as measured with MRS (Kolodny et al., 2020b). Thus, the fMRI undershoot may reflect an inhibitory process that is not captured by MRS measurements of GABA. Ultimately specifying whether this lack of undershoot in ASD has a neural basis (as we modeled in Figure 6A), or a hemodynamic origin (Chen and Pike, 2009) will require future research. However, whatever the underlying cause, a difference in undershoot size has significant implications for the design and interpretation of fMRI experiments that include ASD participants. As our modeling demonstrates, a small difference in undershoot size can manifest as a large difference in the sustained response in some experimental designs (Figure 6C).

A reasonable question is why these differences in sustained and offset response amplitude have not been more commonly observed in previous autism-related fMRI studies. One reason is that the block-lengths in the experimental design are critical for undershoot differences to manifest. For comparison, we used the same model as presented in Figure 6 with a different—but completely plausible—alternative experimental design: 5 individual trials with a 10 s baseline period (Figure 7). With this experimental design, even though there is a difference in undershoot size, there are no differences in the predicted fMRI timecourses; there is not sufficient time for the undershoot differences to accumulate in the sustained response and the post-stimulus effects get truncated by the shorter rest period. Overall, this modeling exercise emphasizes that differences in fMRI response between groups can emerge due to an interaction between neural (or hemodynamic) response components that occur after a stimulus ends and the particular timing of events within the experimental paradigm.

It is important to emphasize that while some of the differences between ASD and NT participants in sustained response could be due to differences in the undershoot, the increase in sustained response in ASD could also reflect disrupted neural adaptation. This possibility was explored in more detail in a previous publication with a subset of these data (Millin et al., 2018). These previous analyses focused on the differences in the auditory and visual cortex and compared different stimulation protocols (fixed vs. randomized-interval). Importantly, those analyses also accounted for and ruled out differences in undershoot size as a single explanation for the change in adaptation—for example, by analyzing sub-groups with equivalent undershoot amplitudes. While disruption in neural adaptation in ASD certainly has implications for overall response magnitude, it does not specifically relate to an increase in neural excitability in this disorder, as typically defined.

The increases in sustained and offset responses observed outside of primary sensory and motor areas (e.g., in middle temporal and posterior parietal; Figures 4C,D) are more difficult to interpret concerning an increase in neural excitability. In the ASD participants, the timecourses in the regions monotonically increase and also exhibit a transient increase after the termination of the stimulation block. While it is clear that an undershoot explanation cannot be easily applied to these regions, whether to interpret this pattern as an increase in neural excitability is questionable. Again, presumably, an increase in excitability would manifest just as much or more at the beginning of a stimulation block as at the end. Ultimately, future research will be required to assign a functional role to these increased responses in ASD.

Though sensory symptoms are common in ASD (Brown and Dunn, 2002; Rogers and Ozonoff, 2005; Ben-Sasson et al., 2009), they have a highly heterogeneous presentation and can include hyperresponsiveness (over-reactivity to sensory stimuli), hyporesponsiveness (under-reactivity to sensory stimuli), and sensation seeking (craving/fascination with certain stimuli; Rogers and Ozonoff, 2005; Ben-Sasson et al., 2009). A previous fMRI experiment using a paradigm similar to the one presented here found response magnitude differences between ASD and NT participants that correlated with sensory over-responsiveness symptoms (Green et al., 2013). Green et al. (2013) did not examine separate components (e.g., transient, sustained, offset) of the fMRI response. Possibly the differences in offset response contributed to their observed fMRI group difference. However, other important methodological differences exist between these studies, such as the active button press in the current experiment vs. passive stimulation in (Green et al., 2013). One speculative implication of our findings is that the inhibitory process that is believed to underlie the fMRI undershoot is disrupted (or absent) in some individuals with ASD and may contribute to sensory symptoms. Future research that modulates offset strength through stimulus manipulations (Mullinger et al., 2017) and associated perceived sensory reactivity may help clarify the role of different neural response components contributing to sensory symptoms in ASD.

Finally, care should be used in interpreting our results and their implications for a change in neural properties such as E/I. Our results strongly suggest that there are not widespread changes in cortical excitability in ASD leading to larger amplitude transient neural responses. However, the link between neural excitability and E/I balance has recently been called into question (Antoine et al., 2019). Specifically, four mouse models of autism were shown to have an increase in E/I ratios but no change in sensory-evoked firing rates suggesting that E/I increases are a compensatory mechanism serving to stabilize neural response magnitudes. Thus, for the interpretation of our results, a lack of difference in transient response magnitudes cannot be used to infer equivalent E/I in individuals with ASD. Overall, while there does not appear to be an obvious increase in neural excitability in ASD, there are clear increases in specific components of the neural response—such as the offset response—that may be important for considering which neural circuits contribute to ASD.

## Data Availability Statement

The datasets generated for this study are available on request to the corresponding author.

## Ethics Statement

The studies involving human participants were reviewed and approved by University of Washington Institutional Review Board. The patients/participants provided their written informed consent to participate in this study.

## Author Contributions

SM designed the experiments, analyzed the data, and wrote the manuscript. TK and M-PS assisted with data collection and analysis and edited the manuscript. JG and RB oversaw clinical assessments and edited the manuscript. RB assisted with experimental design.

## Conflict of Interest

The authors declare that the research was conducted in the absence of any commercial or financial relationships that could be construed as a potential conflict of interest.

## Footnotes

^1^https://support.brainvoyager.com/brainvoyager/available-tools/88-matlab-tools-bvxqtools.
