# Supplementary material for: Late fMRI Response Components Are Altered in Autism Spectrum Disorder
Source: Front Hum Neurosci. 2020 Jun 30;14:241. doi: 10.3389/fnhum.2020.00241 (PMC7338757; doi:10.3389/fnhum.2020.00241)
Supplement: Supplementary file 1 [file Data_Sheet_1.PDF]

Supplementary Material

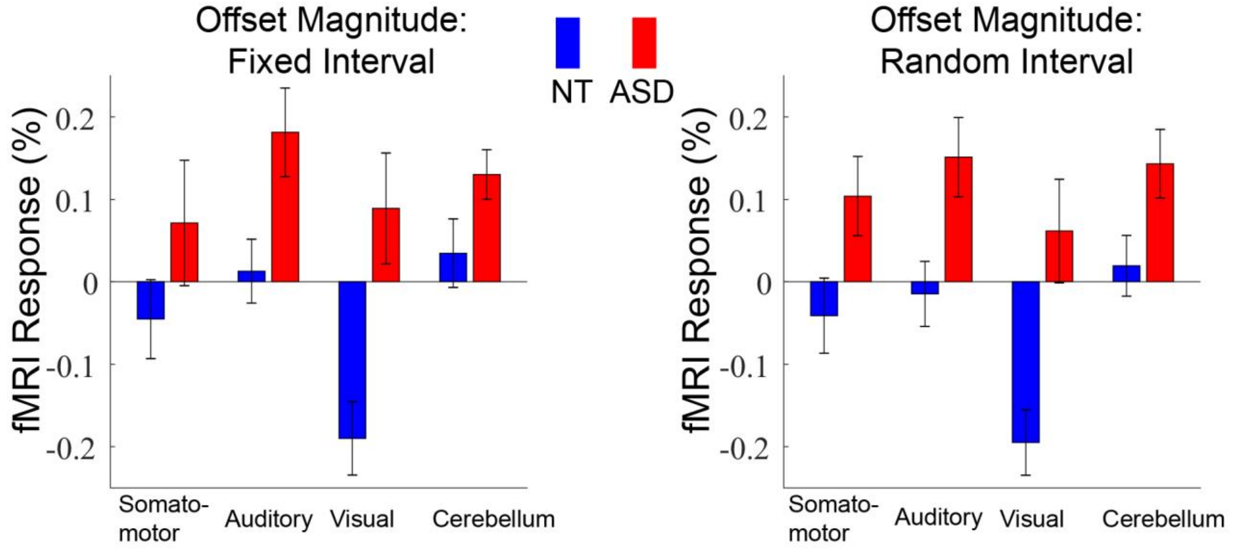

Supplementary Figure 1: Comparison of offset magnitude for both stimulus timing conditions. The individual stimulus events in a block occurred within one of two timing conditions: fixed- or randomized- interval. We examined whether these two timing conditions affected the magnitude of the fMRI offset response (26-30 seconds post stimulation onset) and the differences between groups. By visual inspection of the above figure, the same pattern of results is observed across all four ROIs and between the ASD (red) and NT control (blue) groups in both timing conditions. And, this same pattern is observed in the main results, Fig. 2B, that averages over the timing conditions. To statistically assess any potential differences or interactions we used a mixed ANOVA (group = between subjects; timing condition = within subjects). For all ROIs there was a significant main effect of group (except in the Somato-motor ROI were there was only a trend: $F_{1,46} = 3.85$, $p = 0.056$), no within-subject main effect of timing condition (smallest p-value = 0.41), and no group x timing interaction (smallest p-value = 0.65). Thus, we can conclude that timing condition does not influence the magnitude of the fMRI offset response.

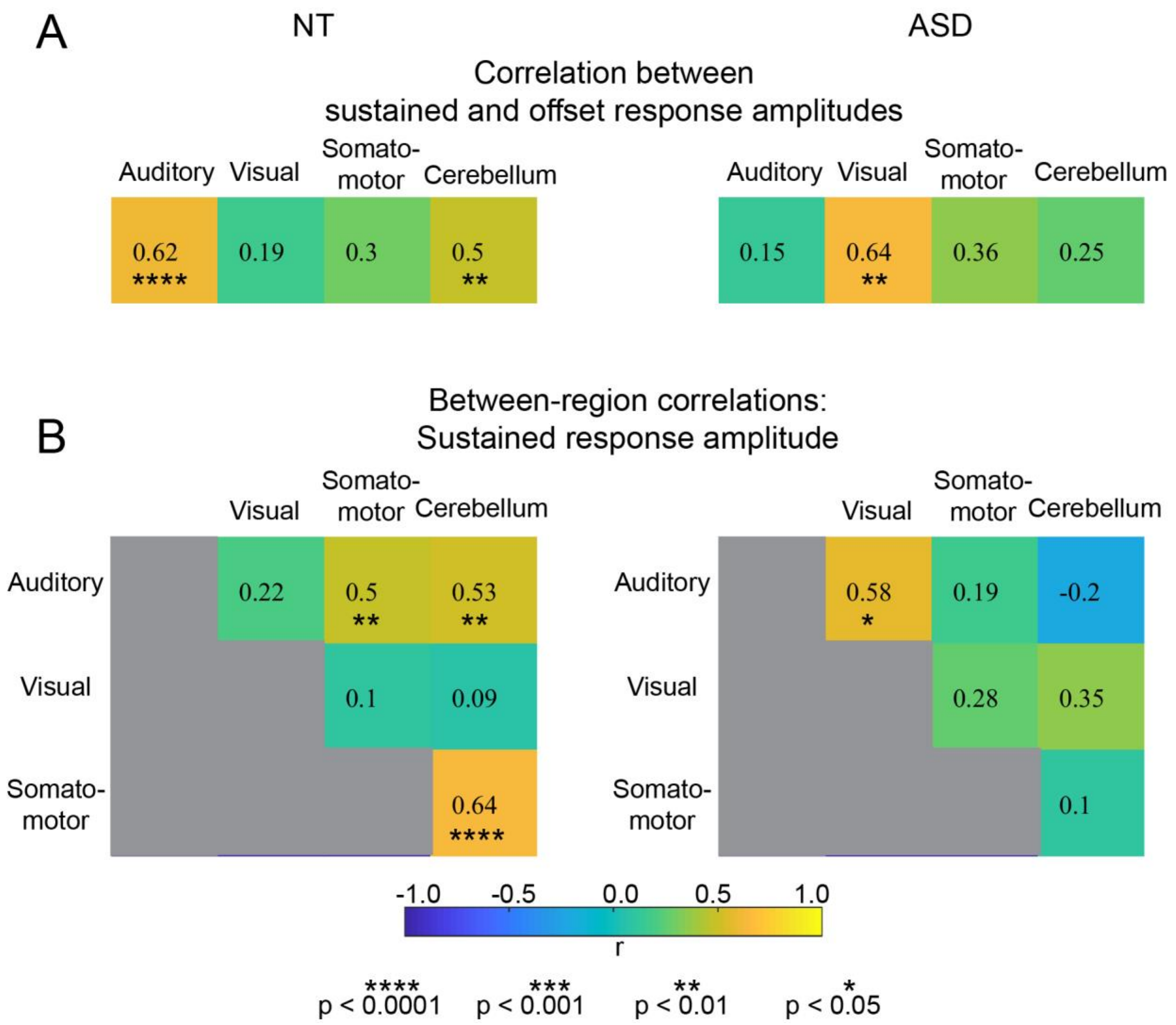

Supplementary Figure 2: Sustained response correlations. The sustained response magnitude can be affected by the transient response magnitude, the offset response (undershoot) magnitude, or some combination of two. Thus, the correlation values for the sustained response are more difficult to interpret but, for completeness, are shown here. (**A**) Correlation values between individual differences in the sustained and offset response. These values can be compared to Fig. 3A in the main manuscript that depicts correlations between transient and offset response amplitudes. (**B**) Correlations between individual differences in sustained response amplitude between regions.

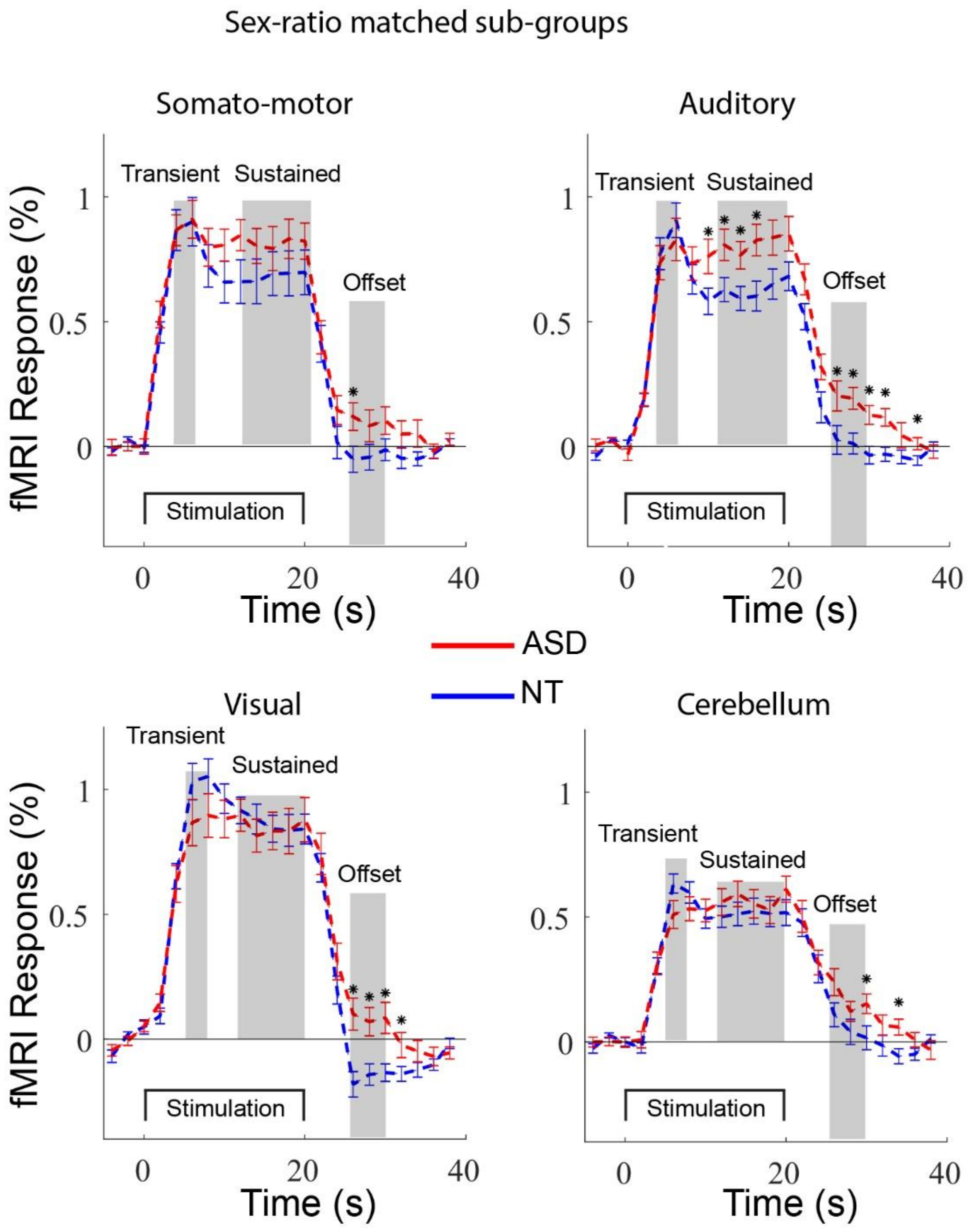

Supplementary Figure 3. Controlling for sex differences in the groups. There is a strong male bias in ASD prevalence (C.D.C, 2014, Werling & Geschwind, 2013) which also manifested in our sample; 5/18 (28%) of the ASD participants were female and 14/32 (44%) of the NT participants were female. To examine whether this difference in sex ratio affected our results, we reduced the NT sample by choosing a random subgroup to reflect the same male female ration as in the ASD group (7/25, 28%). The averaged timecourses are very similar to the full dataset presented in Figure 2. Thus, differences in sex-ratio are unlikely contributing to our results.

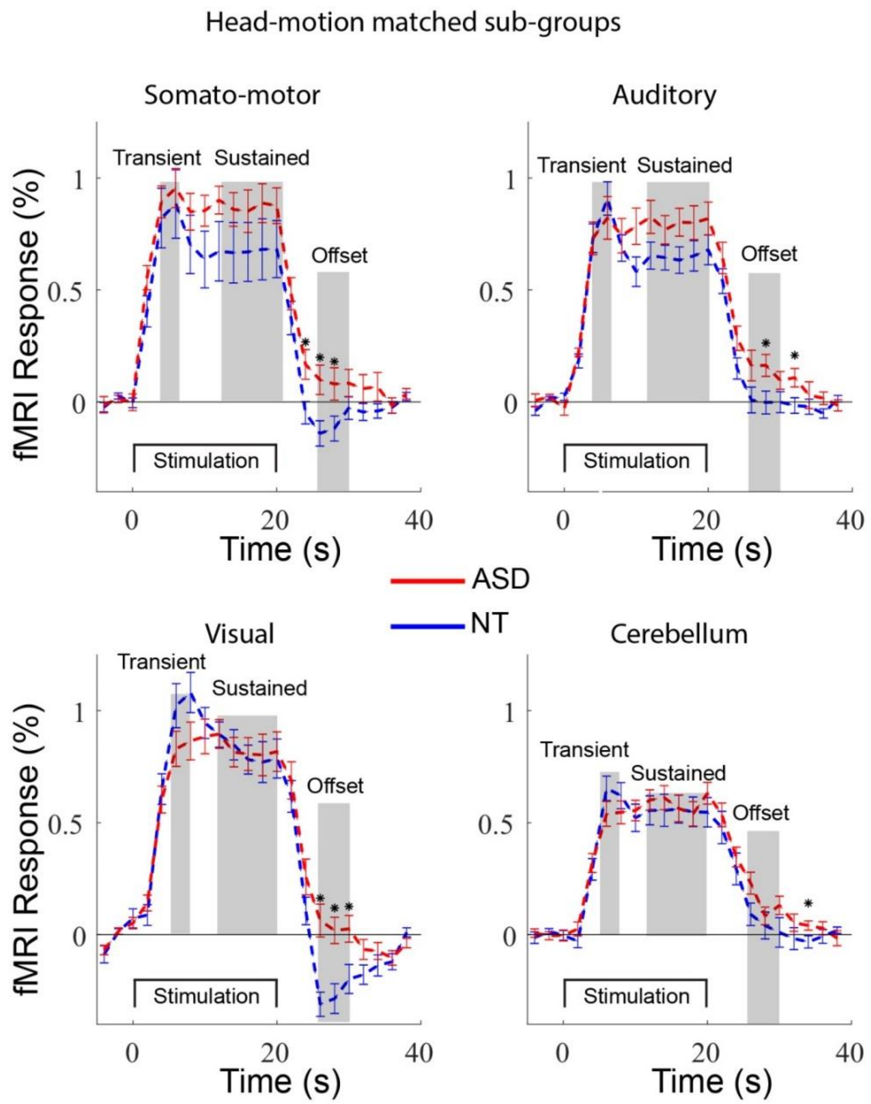

Supplementary Figure 4: Head motion analyses. Even after our data cleaning procedure (see Methods), there was more head motion in the subjects with ASD compared to NT. Specifically, mean framewise displacement was 0.06 mm (std = 0.05) in the NT group and 0.09 mm (std = 0.02) in the ASD group ($t_{48} = 3.06$, $p = 0.004$). To further examine any possible role of head motion in our results, we formed ASD and NT groups matched for head motion. First, we chose the sixteen NT subjects with the most head-motion. Using a least-to-most sorted group of ASD subjects, we added ASD subjects to our prospective group until head-motion was approximately equal to that in the new NT group. This yielded a group of fifteen ASD subjects. Average head motion for these groups was nearly identical: NT = 0.07 mm (0.02); ASD = 0.07 (0.03), $t_{29} = 0.38$, $p = 0.71$. We then performed the ROI analyses on these head-motion equivalent groups. The averaged timecourses are very similar to the full dataset presented in Figure 2. For example, a prominent undershoot difference remains in the offset response in all of the ROIs except the cerebellum. Thus, differences in head motion are unlikely to be driving the observed group differences in offset response.
